# Proteinuria and risk of ocular motor cranial nerve palsy: a nationwide population-based study

**DOI:** 10.1038/s41598-024-62576-0

**Published:** 2024-05-26

**Authors:** Juha Lee, Kyungdo Han, Juhwan Yoo, Kyung-Ah Park, Sei Yeul Oh

**Affiliations:** 1 Department of Ophthalmology, Kangwon National University Hospital, Chuncheon, Republic of Korea; 2 Department of Statistics and Actuarial Science, Soongsil University, Seoul, Republic of Korea; 3 Department of Biomedicine and Health Science, The Catholic University of Korea, Seoul, Republic of Korea; 4 Department of Ophthalmology, Samsung Medical Center, Sungkyunkwan University School of Medicine, 81 Irwon-ro, Gangnam-gu, Seoul, 06351 Republic of Korea

**Keywords:** Proteinuria, Ocular motor cranial nerve palsy, Sixth cranial nerve palsy, Fourth cranial nerve palsy, Third cranial nerve palsy, Ocular motility disorders, Epidemiology

## Abstract

Understanding the association between dipstick-detected proteinuria and oculomotor cranial nerve palsy (CNP) could have significant implications for understanding the mechanism of CNP development and for developing preventive strategies against CNP development in patients with proteinuria. This study aimed to determine the relationship between dipstick-determined proteinuria and ocular motor CNP using National Sample Cohort (NSC) database from Korea’s National Health Insurance Service (NHIS). A nationwide population-based cohort study was conducted using data from the NSC database of Korea’s NHIS. These data were collected from 2009 to 2018. A one-year time lag was established to prevent a situation in which the causal link was inverted. Participants aged 20 years or more who were diagnosed with proteinuria in 2009 were included. Individuals with specific pre-existing CNP, missing data, and those who were newly diagnosed with CNP or who died within one year of being tested were excluded. The study population was classified into six groups according to the degree of proteinuria (negative, trace, or between 1 + and 4 +) based on the urine dipstick test. A Cox proportional hazard regression analysis was performed to determine the linkage between the degree of proteinuria and ocular motor CNP. A total of 5,807 (0.14% of subjects) with ocular motor CNP were assigned to the ocular motor CNP group and 4,047,205 subjects were assigned to the control group. After full adjustment of comorbidities, hazard ratios (HRs) for 1 + , 2 + , 3 + and 4 + proteinuria groups were 1.449 (95% confidence interval [CI] 1.244–1.687), 2.081 (1.707–2.538), 1.96 (1.322–2.904), and 3.011 (1.507–6.014), respectively, for developing ocular motor CNP compared to the proteinuria-negative group. In subgroup analysis, the HR of patients with proteinuria for the development of ocular motor CNP was higher in the younger age group (less than 40 years) (*P* = 0.0242) and the group with DM (*P* = 0.04). Our population-based cohort study demonstrated a significant association between proteinuria and the incidence of CNP, suggesting that urine protein level could be a new clinical marker for predicting the development of CNP.

## Introduction

Third, fourth, and sixth cranial nerve palsies (CNP) are known to affect extraocular muscles and cause symptoms of diplopia with or without abnormal head posture. They are common disease entities encountered in neuro-ophthalmology clinics. Along with paralysis of the facial nerve, paralysis of ocular motor cranial nerves (CNs) including CN3 (oculomotor nerve), CN4 (trochlear nerve), and/or CN6 (abducens nerve) is one of the most frequent mononeuropathies of the CN^1^. Many previous reports have revealed that ocular motor CNP (OCNP) has close associations with diseases that can causing vasculopathy, such as diabetes mellitus, hyperlipidemia, hypertension, and several circulatory diseases that can increase with age and lead to atherosclerotic changes^2–4^. Other conditions including aneurysm, trauma, neoplasm, brain stem infarction, infection, inflammation, sinus thrombosis, and multiple sclerosis can also cause the development of OCNP^5–8^. Although heterogeneous mechanisms might be involved in the development of OCNP^5–8^, microvascular ischemia caused by atherosclerosis is the most widely postulated mechanism for OCNP^1^.

Chronic kidney disease (CKD) is a worldwide public health problem. It has been estimated that approximately 10–15% of adults in developed countries are affected by CKD^9–11^. CKD is also known to cause neurological complications in peripheral and central nervous systems^12^. Complications in peripheral nervous system include somatic neuropathy, myopathy, and cranial neuropathy^12^. Among those with cranial neuropathies, acoustic and olfactory nerves are frequently involved^12^, whereas optic neuropathy is rarely reported^13^. Cognitive dysfunction, delirium, encephalopathy, dementia, focal stroke-related symptoms, and cortically originated abnormal movements such as cortical myoclonus, asterixis, and epileptic seizures are central nervous system disorders known to be associated with CKD^12^.

Proteinuria is a biomarker of kidney damage that can predict early stages of CKD^14^. Its severity is correlated with a rapid decline in renal function^15^. Proteinuria can also predict patients at increased risk of adverse clinical outcomes regardless of the estimated glomerular filtration rate (eGFR)^16^. Proteinuria is known to manifest endothelial dysfunction and systemic inflammation^17^. It has been recently shown that proteinuria is associated with metabolic syndrome, hypertension, diabetes, fatty liver, Parkinson’s disease, and Crohn’s disease. In addition, proteinuria is associated with a worsening disease course in stroke, non-Hodgkin’s lymphoma, and endocarditis^18–22^. In recent years, the use of human urine samples to search for biomarkers has gained increasing interest due to its convenient and non-invasive collection with stable compositions^23^. Although urine protein-to-creatinine ratio (PCR) and urine albumin-to-creatinine ratio (ACR) are used as quantitative tests for proteinuria, their application in public health screening is prohibitively expensive. In addition, it takes a significant amount of time to confirm their results. However, a urine dipstick test is a relatively simple, fast, and inexpensive method for screening proteinuria in public healthcare systems.

To the best of our knowledge, studies examining the association between proteinuria and the development of OCNP are uncommon. Thus, the purpose of this study was to determine the association of dipstick-determined proteinuria with OCNP using a large general population.

## Results

### Baseline clinical characteristics

A total of 4,053,012 subjects were eligible for this study. They were tracked for 33,305,711.65 person-years. According to the degree of proteinuria, they were divided into six groups: negative (n = 3,857,777, 95.18%), trace (n = 92,700, 2.29%), 1 + (68,923, 1.70%), 2 + (25,728, 0.63%), 3 + (6577, 0.16%), and 4 + (1307, 0.03%). Baseline characteristics were compared among subjects with varying degrees of proteinuria (Table 1). Age and waist measurements were positively correlated with the severity of proteinuria (both *P* < 0.001). Additionally, the group with severe proteinuria had higher rates of co-morbid conditions such as hypertension, diabetes, and dyslipidemia (all *P* < 0.001). However, individuals with higher levels of proteinuria had significantly lower rates of current smoking (*P* < 0.001). Other variables including gender, alcohol consumption, regular exercise, low income, and eGFR demonstrated significant differences according to proteinuria severity (all *P* < 0.0001).

**Table 1:** Baseline characteristics of patients with ocular motor cranial nerve palsy and controls.

Variables	Groups of proteinuria						*P* value
	Negative	Trace	+ 1	+ 2	+ 3	+ 4	
	(N = 3,857,777)	(N = 92,700)	(N = 68,923)	(N = 25,728)	(N = 6577)	(N = 1307)	
Age, years	46.87 ± 14.02	48.75 ± 14.18	51 ± 14.42	52.71 ± 14.34	54.1 ± 13.77	55.42 ± 14.03	< .0001
Sex, male, N (%)	2,124,367 (55.07)	50,758 (54.76)	37,479 (54.38)	14,446 (56.15)	3824 (58.14)	752 (57.54)	< .0001
Smoking status, N (%)							< .0001
Non-smoker	2,289,508 (59.35)	55,080 (59.42)	40,943 (59.4)	15,219 (59.15)	3798 (57.75)	801 (61.29)	
Ex-smoker	553,930 (14.36)	13,875 (14.97)	10,710 (15.54)	4194 (16.3)	1147 (17.44)	219 (16.76)	
Current smoker	1,014,339 (26.29)	23,745 (25.61)	17,270 (25.06)	6315 (24.55)	1632 (24.81)	287 (21.96)	
Drinking amount, N (%)^a^							< .0001
None	1,980,034 (51.33)	48,070 (51.86)	37,085 (53.81)	14,450 (56.16)	3836 (58.32)	826 (63.2)	
Mild	1,572,595 (40.76)	36,455 (39.33)	25,374 (36.81)	8816 (34.27)	2128 (32.36)	372 (28.46)	
Heavy	305,148 (7.91)	8175 (8.82)	6464 (9.38)	2462 (9.57)	613 (9.32)	109 (8.34)	
Regular exercise, N (%)^b^	699,134 (18.12)	17,623 (19.01)	13,033 (18.91)	4942 (19.21)	1196 (18.18)	244 (18.67)	< .0001
Low income, N (%)^c^	673,523 (17.46)	15,090 (16.28)	12,265 (17.8)	4671 (18.16)	1261 (19.17)	247 (18.9)	< .0001
Waist Circum	80.17 ± 9.4	81.13 ± 9.9	82.27 ± 10.62	83.21 ± 10.05	84.11 ± 13.19	83.86 ± 10.22	< .0001
Dyslipidemia, N (%)^d^	683,658 (17.72)	21,362 (23.04)	19,304 (28.01)	8987 (34.93)	2877 (43.74)	619 (47.36)	< .0001
*TG	112.27 (112.21–112.34)	114.92 (114.48–115.36)	123.68 (123.11–124.25)	133.85 (132.83–134.88)	146.39 (144.2–148.61)	148.58 (143.67–153.66)	< .0001
HDL	56.57 ± 33.14	55.9 ± 29.37	55.28 ± 25.31	54.51 ± 28.96	53.92 ± 30.46	53.19 ± 25.29	< .0001
HTN, N (%)^e^	1,006,089 (26.08)	32,799 (35.38)	31,292 (45.4)	14,539 (56.51)	4348 (66.11)	899 (68.78)	< .0001
DM, N (%)^f^	313,571 (8.13)	13,280 (14.33)	14,986 (21.74)	7689 (29.89)	2484 (37.77)	549 (42)	< .0001
eGFR^g^ (mL/min/1.73m^2^)	87.85 ± 45.34	83.63 ± 32.45	81.9 ± 35	77.74 ± 37.53	72.3 ± 32.55	72.66 ± 52.18	< .0001

*CI* Confidence interval, *BMI* Body mass index, *HTN* Hypertension, *DM* Diabetes mellitus, *AST* Aspartate aminotransferase, *ALT* Alanine aminotransferase.

^a^Drinking amount: Individuals who consumed more than 30 g of alcohol per day were defined as heavy drinkers.

^b^Regular exercise: Regular physical activity was defined as an individual doing high-intensity exercise for at least 20 min three times per week or at least 30 min of moderate-intensity exercise five times per week.

^c^Low income: The low-income level was defined as the bottom 20% of the total population.

^d^Dyslipidemia: Dyslipidemia was defined as a total cholesterol level of 240 mg/dL or higher, or at least once per year for prescription of a lipid-lowering drug under ICD-10-CM code E78.

^e^HTN: Hypertension was defined as being prescribed at least once per year for antihypertensive drugs under ICD-10-CM codes I10-I13, I15, or BP ≥ 140/90 mmHg.

^f^DM: DM was defined as fasting glucose ≥ 126 mg/dL, or at least once per year for a prescript.

^g^eGFR: estimated glomerular filtration rate.

### Incidence and risk of OCNP according to proteinuria level by dipstick.

Among eligible subjects, 5,807 developed OCNP. They were assigned to the OCNP group. Remaining subjects (n = 4,047,205) were assigned to the control group. The incidence of OCNP was 0.14%. The average duration of follow-up was 8.22 ± 0.94 years.

The risk of developing OCNP was significantly higher in the proteinuria-positive group than in the proteinuria-negative group (crude hazard ratios [HRs] : 1.848, 2.939, 2.971, and 4.89 in 1 + , 2 + , 3 + and 4 + proteinuria groups, respectively, Model 1 in Table 2). A higher degree of proteinuria on the dipstick test was associated with a higher risk of developing OCNP (crude HR: 4. 89; 95% CI 2.448-9.769 in the 4+ Proteinuria group; Table 2).

**Table 2:** Incidence of ocular motor cranial nerve palsy according to the proteinuria.

	N	CNP	DURATION	Incidence rate (per 1000 person-years)	Hazard ratios (95% confidence intervals)			
					Model 1	Model 2	Model 3	Model 4
Dipstick urinalysis								
Negative	3,857,777	5340	31,732,491.05	0.16828	1.000	1.000	1.000	1.000
Trace	92,700	162	756,173.1	0.21424	1.274 (1.09,1.489)	1.162 (0.994,1.359)	1.164 (0.995,1.361)	1.132 (0.968,1.324)
+ 1	68,923	172	554,039.99	0.31045	1.848 (1.588,2.151)	1.524 (1.309,1.774)	1.524 (1.309,1.775)	1.449 (1.244,1.687)
+ 2	25,728	100	202,846.67	0.49298	2.939 (2.411,3.582)	2.25 (1.845,2.742)	2.246 (1.842,2.738)	2.081 (1.707,2.538)
+ 3	6577	25	50,323.23	0.49679	2.971 (2.006,4.402)	2.17 (1.465,3.215)	2.161 (1.458,3.201)	1.96 (1.322,2.904)
+ 4	1307	8	9837.61	0.81321	4.89 (2.448,9.769)	3.384 (1.695,6.758)	3.359 (1.682,6.707)	3.011 (1.507,6.014)
eGFR (mL/min/1.73m^2^)								
90 ≤	1,597,917	1930	13,179,220.91	0.14644	1.000	1.000	1.000	1.000
60–90	2,176,989	3340	17,893,387.6	0.18666	1.274 (1.205,1.348)	0.988 (0.934,1.046)	0.986 (0.932,1.044)	0.974 (0.92,1.031)
< 60	278,106	537	2,233,103.14	0.24047	1.644 (1.495,1.809)	0.969 (0.878,1.069)	0.965 (0.874,1.064)	0.889 (0.805,0.982)

eGFR, estimated glomerular filtration rate.

Model 1 is not adjusted for any covariates.

Model 2 is adjusted for age and sex.

Model 3 is adjusted for age, sex, smoking status, alcohol consumption, regularity of physical activity, and status of income.

Model 4 is adjusted for age, sex, smoking status, alcohol consumption, regularity of physical activity, status of income, metabolic diseases including hypertension, diabetes, and dyslipidemia, and glomerular filtration rate.

**Figure 1:**
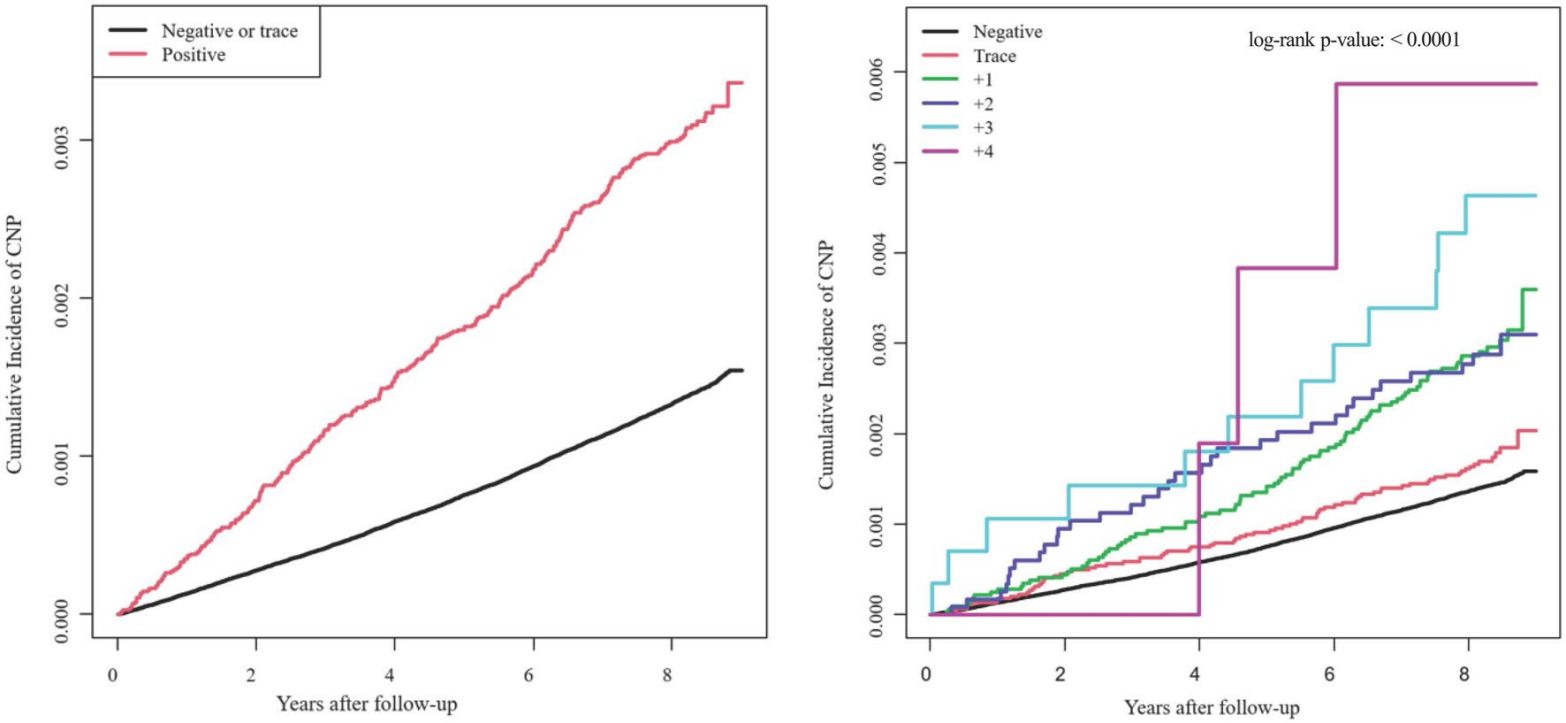
Kaplan–Meier curves for ocular motor cranial nerve palsy (OCNP) incidence according to the degree of dipstick proteinuria. The incidence probability of OCNP according to the presence of proteinuria (Left) and levels of proteinuria (Right) are shown.

When assessing the independent relationship between proteinuria level and OCNP, we accounted for those potential confounding variables. After adjusting for age, sex, income level, smoking status, alcohol consumption status, exercise level, and comorbidities (DM, hypertension, and dyslipidemia) in model 4, the presence of proteinuria was still associated with the development of CNP. The incidence of OCNP increased with increasing urine protein levels. With the group of no proteinuria as a reference, the adjusted hazard ratio (HR) was 1.449 (95% confidence interval [CI]: 1.244–1.687) for the + 1 proteinuria group, 2.081 (95% CI 1.707–2.538) and 1.96 (95% CI 1.322-2.904) for both + 2 and + 3 proteinuria groups, and 3.011 (95% CI 1.507–6.014) for the + 4 proteinuria group (Table 2). The cumulative incidence of OCNP is displayed in Fig. 1. It increased with increasing level of proteinuria. The degree of proteinuria detected by the dipstick test was related to an increased likelihood of OCNP development (Fig. 1).

### Risk of OCNP in subgroup analysis according to age, sex, diabetes mellitus, hypertension, dyslipidemia, and metabolic syndrome

We conducted subgroup analyses to determine effects of dipstick-positive proteinuria (1 + proteinuria) on the development of OCNP stratified by age, sex, metabolism disorder, DM, hypertension, dyslipidemia, BMI, and history of cardiovascular disease (Table 3). The analysis was also adjusted for other variables such as age, sex, smoking status, drinking amount, regularity of physical activity, status of income, metabolic syndrome, and eGFR. The impact of dipstick-positive proteinuria on onset of OCNP varied significantly according to age (*P* = 0.0242) and DM (*P* = 0.04). Those with proteinuria in the age group of less than 40 years had a higher HR of 1.928 (95% CI 1.22–3.048) for the development of OCNP compared to other age groups, including the age group of 40–64-years (HR: 1.835, 95% CI 1.588–2.12) and the age group of 65 years or more (HR:1.335, 95% CI 1.077–1.655). Those with proteinuria in the DM group had a higher HR of 1.727 (95% CI 1.467–2.033) for the development of OCNP compared to the group without DM (HR:1.306, 95% CI 1.103–1.547).

**Table 3:** Subgroup analysis of the risk of ocular motor cranial nerve palsy among people with dipstick-positive proteinuria.

Subgroup	Negative or trace on dipstick				Positive on dipstick				Adjusted HR^a^ (95% CI)	*P* for interaction
	N	CNP	Duration	Rate	N	CNP	Duration	Rate		
Age, years										
< 40	1,259,710	594	10,468,368.84	0.05674	20,426	19	169,175.76	0.11231	1.928 (1.22,3.048)	.0242
40–64	2,189,179	3359	18,122,349.15	0.18535	60,511	197	492,362.15	0.40011	1.835 (1.588,2.12)	
≥ 65	501,588	1549	3,897,946.17	0.39739	21,598	89	155,509.6	0.57231	1.335 (1.077,1.655)	
Sex										
Male	2,175,125	3614	17,802,251.48	0.20301	56,501	205	444,157.55	0.46155	1.616 (1.403,1.863)	.8355
Female	1,775,352	1888	14,686,412.67	0.12855	46,034	100	372,889.95	0.26818	1.746 (1.427,2.137)	
METS										
No	3,144,475	3579	25,928,445.66	0.13803	61,177	115	493,563.8	0.233	1.441 (1.197,1.736)	.0594
Yes	806,002	1923	6,560,218.49	0.29313	41,358	190	323,483.7	0.58736	1.855 (1.598,2.154)	
Diabetes mellitus										
No	3,623,626	4293	29,878,768.24	0.14368	76,827	139	622,216.96	0.22339	1.306 (1.103,1.547)	.04
Yes	326,851	1209	2,609,895.91	0.46324	25,708	166	194,830.55	0.85202	1.727 (1.467,2.033)	
Hypertension										
No	2,911,589	3164	24,084,256.64	0.13137	51,457	100	420,767.21	0.23766	1.592 (1.305,1.944)	.7808
Yes	1,038,888	2338	8,404,407.51	0.27819	51,078	205	396,280.3	0.51731	1.691 (1.465,1.952)	
Dyslipidemia										
No	3,245,457	4099	26,711,308.18	0.15346	70,748	179	566,454.63	0.316	1.594 (1.372,1.853)	.4512
Yes	705,020	1403	5,777,355.97	0.24284	31,787	126	250,592.87	0.50281	1.769 (1.473,2.126)	
BMI										
< 25	2,667,217	3295	21,913,399.37	0.15036	60,001	156	474,800.39	0.32856	1.666 (1.417,1.958)	.9952
≥ 25	1,283,260	2207	10,575,264.78	0.20869	42,534	149	342,247.12	0.43536	1.658 (1.403,1.959)	
CVD										
No	2,409,512	3493	19,834,412.88	0.17611	66,263	215	526,880.25	0.40806	1.721 (1.498,1.977)	.677
Yes	100,787	275	795,174.01	0.34584	5245	25	38,062.81	0.65681	1.631 (1.079,2.465)	
Missing	1,440,178	1734	11,859,077.26	0.14622	31,027	65	252,104.44	0.25783	1.511 (1.179,1.937)	

*CNP* Ocular motor cranial nerve palsy, *CI* Confidence interval, *HR* Hazard ratio, *METS* Metabolic syndrome, *BMI* Body mass index (kg/m^2^), *eGFR* Estimated glomerular filtration rate.

^a^Age, sex, smoking status, alcohol consumption, regularity of physical activity, status of income, metabolic diseases including hypertension, diabetes, and dyslipidemia were adjusted for.

## Discussion

Results of this study revealed that the incidence of OCNP among Koreans was positively associated with the presence of proteinuria. These outcomes were significant with or without adjustment for various confounders, including age, sex, smoking status, drinking amount, regularity of physical activity, the presence of metabolic syndrome, and eGFR. Especially, those with proteinuria in the age group of less than 40 years old had a higher risk of developing OCNP compared to those with proteinuria in other age groups, including the age group of 40–64 years and the age group of 65 years or more.

Proteinuria is known to be more likely to occur with aging or with comorbidities such as DM and HTN^24,25^. An epidemiological study based on the U.S. Third National Health and Nutrition Examination Survey (NHANES III) aimed to determine the association of renal insufficiency with albuminuria in whole population and in patients with DM or HTN showed similar results to ours^25^. In the whole population of that study, the prevalence of albuminuria was higher in older participants: 19.1% in participants 60 years of age or older vs. < 6% in those under 40 years of age. Moreover, the prevalence of albuminuria was 34.2% in a diabetic population, 14.5% in a non-diabetic hypertensive population without DM, and 5.1% in a non-diabetic non-hypertensive population^25^. However, when they observed the relationship between presence of albuminuria and renal insufficiency, 88.4% of patients with renal insufficiency showed albuminuria in those under 40 years of age, whereas only 43.6% of patients showed albuminuric renal insufficiency in those over 60–79 years of age^25^. In the Heart Outcomes and Prevention Evaluation (HOPE) study, two-thirds of elderly patients with renal insufficiency showed no signs of albuminuria^26^. The above studies suggest that proteinuria could reflect renal function impairment better in people under 40 years of age than in elder age. In our study, proteinuria showed a greater impact on the occurrence of CNP in people under the age of 40. Because the adjusted OR in our study was adjusted for several factors, including DM and HTN, it was hard to say that the difference in CNP occurrence was simply due to an increase of underlying disease caused by aging. We found that the occurrence of CNP was increased 1.928 (CI 1.22–3.048) times with proteinuria in young age groups (younger than 40 years). Our results suggest that caution should be exercised when proteinuria is positive in people under 40 years of age.

In sub-analysis, we classified the degree of dipstick test for proteinuria from + 1 to + 4 as one group compared to negative and trace ( ±). According to the study of Iseki et al^27^., even a slight increase in dipstick urinalysis for proteinuria was an independent risk factor for ESRD. When the proteinuria results based on dipstick urinalysis were analyzed by dividing them to negative, trace, + 1, + 2, and ≥  + 3, the incidence of end-stage renal disease (ESRD) rose significantly and consistently as the degree of proteinuria increased. Although + 1 was not cost-effective, + 1 was found to significantly increase the risk of ESRD compared to negative (adjusted odds ratio: 1.93 (95% CI 1.53–2.41, *P* < 0.001) in men and 2.42 (95% CI 1.91–3.06, *P* < 0.001) in women)^27^. Even a slight increase in the degree of proteinuria such as proteinuria (trace; ±) caused a change in the adjusted odds ratio (95% CI)^27^. Proteinuria has been widely used as a biomarker for kidney disease in clinical practice^28^. The extent of renal dysfunction is also frequently estimated by measuring serum creatinine and calculating the eGFR^29^. Both eGFR and proteinuria are known to be pivotal markers for the assessment of risk such as cardiovascular risk including mortality and cardiovascular events as well as overall prognosis of CKD^30^. Interpretation of results of eGFR must be done with caution because it is affected by age, sex, and lean muscle mass. In contrast, proteinuria and albuminuria are complementary indices of renal function that can assess damage to the renal filtration barrier^31–33^. Proteinuria is not simply a subsequent outcome of kidney damage. It can also pathologically produce renal tubulointerstitial damages, resulting in progressive renal function loss^34^. Decreased eGFR or the presence of overt proteinuria is also independently related to increased all-cause mortality^35–37^. In our study, those with more than degree of + 1 in dipstick test for proteinuria showed an increase in the incidence of OCNP. As to eGFR, those with eGFR more than 90 mL/min/1.73 m^2^ showed an increased incidence of OCNP than those with eGFR < 60 mL/min/1.73 m^2^. Two issues can emerge when observing association between eGFR and risk of diseases. The first issue is that a few studies have reported that eGFR lower than 60 mL/min/1.73 m^2^ can be an independent predictor of mortality risk^38,39^. However, Wu et al. have suggested that a lack of distinction below eGFR < 60 mL/min/1.73 m^2^ might overstate the cut-off point of eGFR to predict a result such as mortality^40^. In a prospective longitudinal cohort study that included 821 consecutive patients hospitalized due to acute stroke, eGFR < 45 mL/min/1.73 m^2^ (vs. eGFR ≥ 60 mL/min/1.73 m^2^) was found to be an independent predictor of higher occurrence of death or severe sequelae after acute stoke^41^. In addition, eGFR of < 50 mL/min/1.73 m^2^ (vs. eGFR ≥ 60 mL/min/1.73 m^2^) was significantly associated with the incidence of hypertension (HR: 1.29 (1.03–1.61)) in young to middle aged people (median age: 35 (30–40) years)^42^. Wu et al. have suggested that 45 mL/min/1.73 m^2^ but not 60 mL/min/1.73 m^2^ might be the cut-off point for eGFR as an independent predictor of all-cause mortality for a population in northern China^40^. The second issue is that some results have indicated a U-shaped or J-shaped relationship between eGFR and various diseases. A multicenter population-based stroke registry-based study conducted in Japan involving 1,400,000 individuals has revealed that hazard ratios for all in-hospital death and at-discharge death/disability are higher in those with eGFR < 45 mL/min/1.73 m^2^ (OR: 1.54) and those with eGFR ≥ 90 mL/min/1.73 m^2^ (OR: 1.48) than in the reference group (eGFR = 60–89 mL/min/1.73 m^2^)^43^. Another study has shown U-shaped observational associations of creatinine-based eGFR with CHD and stroke, with participants with eGFR values < 60 or > 105 mL/min/1.73 m^2^ having a higher risk for CHD and stroke than those with eGFR between 60 and 105 mL/min/1.73 m^2^^44^. In our study, there was a significant difference in CNP occurrence between those with eGFR < 60 mL/min/1.73 m^2^ and those with eGFR > 90 mL/min/1.73 m^2^. CNP occurrence might have increased when eGFR levels were above 90 mL/min/1.73 m^2^ rather than decreased when eGFR levels were below 60 mL/min/1.73 m^2^. An elevated eGFR could be a potential risk factor for the development of CNP in certain populations. Further investigation into specific eGFR levels and their impact on OCNP is necessary.

The most widely postulated mechanism in OCNP is microvascular ischemia to the CN due to atherosclerotic risk factors^1^, accounting for 20.7% of CN3 palsies, 18.6% of CN4 palsies, and 17.7% of CN6 palsies^7,45^. Diabetes mellitus, hyperlipidemia, and hypertension are the most prevalent microvascular ischemic conditions leading to atherosclerosis^46^. These conditions are commonly associated with stroke^1^. Isolated OCNP has also been reported to be a risk factor for subsequent stroke in past epidemiological studies^1,47,48^. Clinical data have revealed that patients with CN 3/4/6 palsies and those with CN3 palsies are 2.74 times and 3.69 times more likely to experience a stroke, respectively^1^. This has been explained by a common pathophysiology shared between the underlying disease and atherosclerosis. CKD also shares conventional cardiovascular risk factors such as diabetes, hypertension, obesity, and smoking^29^. A cohort study has postulated that proteinuria, but not eGFR, can be a useful biomarker to predict stroke in CKD^29^. It proposed that CKD and cerebrovascular disease might share similar pathogenic mechanisms^29^. The causal mechanism between CKD and ischemic stroke is as follows. First, both the kidney and brain have microvasculature with low resistance that can be continuously perfused with a high amount of blood^29^. Second, endothelial damage is recognized as a common mediator of small vessel cerebrovascular disease and renal dysfunction of the brain^29,49^. Endothelial damage can induce basement membrane thickening and cellular proliferation, resulting in narrowing of the vascular lumen^5,50^. Albuminuria has been postulated to indicate systemic endothelial dysfunction^51,52^, which affects inflammatory and thrombotic cascades^53,54^ and contributes to atherothrombotic events such as stroke. Third, disruption of the integrity of the blood–brain barrier by uremia might have implications for small vessel disease and lacunar stroke risk^55^. Serum component leakage into and through walls of small cerebral vessels cam lead to neuronal and glial damage, which might be an essential common mechanism for these disparate conditions^55^. The widespread development of OCNPs in proteinuria in this study was presumed to result from a similar micro-ischemic process.

This study has several limitations. Since the dipstick test can respond to proteins other than albumin, low-grade proteinuria does not always indicate albuminuria. Despite the inaccuracy of albuminuria measurement, urine dipstick test is useful for risk stratification due to its low cost and simplicity. Second, because this was a cross-sectional study, there was no evidence of a cause-and-effect relationship between OCNP and proteinuria. Third, the high cut-off value prevented the observation of the impact of a decrease in eGFR on the occurrence of CNP. Further research is required to distinguish eGFR into more specific units to uncover the U-shaped relationship between eGFR and CNP occurrence that might have been concealed. Last, there were numerous confounders between OCNP and proteinuria. Although our study showed significant results after adjusting for these confounders, additional research is required to identify biomarkers that can directly explain this mechanism more directly.

In conclusion, we found that subjects with dipstick-determined proteinuria showed an increased risk of having OCNP in a large general population. Especially, patients with proteinuria in the age group of 40 years or younger have a risk of OCNP development. If the pathophysiological mechanism linking the two diseases is investigated, a method of early OCNP prevention can be proposed.

## Materials and methods

In this cohort study, medical data from Korea’s National Health Insurance Service (NHIS), a national insurer covering approximately 97% of the Korean population under the supervision of the Ministry of Health and Welfare, were analyzed. The NHIS database contains demographic data, disease codes, procedural codes, details on inpatient and outpatient care, and information on prescribed medications. The government recommends regular health checkups at least biannually for enrollees of the NHIS that all Koreans are mandatory to subscribe to^56,57^. In the above database, the NHIS National Sample Cohort (NSC) database of Korea were utilized to investigate the association between chronic kidney disease and OCNP in Korean adults.

Data of 4,233,273 individuals aged 20 years or more who underwent regular health checkups in 2009 were collected. This study assessed a total of 4,053,012 eligible persons after removing those who had previously been diagnosed with OCNP (n = 2872), those with missing data (n = 166,467), and those who were newly diagnosed with OCNP or who died within one year of being tested (n = 10,922). Participants ultimately included in the study population were tracked until December 31, 2018. International Classification of Diseases, Tenth Revision, Clinical Modification (ICD-10-CM) codes were used to identify patients who had recently developed ocular motor CNP. A one-year time lag was established to prevent a situation in which the causal link was inverted.

This study adhered to principles outlined in the Declaration of Helsinki. It was authorized by the Institutional Review Board (IRB) of Samsung Medical Center (IRB no. SMC 2020-09-050). Patients' informed consent was waived because data used in this study were publicly accessible and anonymized in accordance with confidentiality guidelines.

### Definition of OCNP and confounders

This study’s primary outcome was a newly developed OCNP. For identifying OCNP, ICD-10 codes H49.0 (3rd CNP), H49.1 (4th CNP), and H49.2 (6th CNP) were utilized. In this study, those with diagnosis codes of H06.2 (dysthyroid exophthalmos), E05 (thyrotoxicosis), or G70.0 (myasthenia gravis) were excluded^58^. Age, gender, income status, smoking status, regular physical activity, and drinking amount were considered as confounding variables in the relationship between OCNP and proteinuria.

Baseline data including age, findings at physical examination, and laboratory data were collected at the time of diagnosis of proteinuria. Physical examinations including height, weight, body mass index (BMI), blood pressure, and basic laboratory tests were used as covariates. Body mass index (BMI) was calculated by dividing weight (kg) by an individual's height (in meters) squared. Using self-reported questionnaires, information on past medical history and social history (including alcohol consumption and smoking status, and physical activity) were gathered. Information was gathered from the questionnaire completed at the time of proteinuria diagnosis. Lifestyle habits were recorded as either current or past depending on the time of diagnosis. Participants in the study were classified as either nonsmokers, formerly smoking individuals, or currently smoking individuals about their smoking status. Regarding the use of alcohol, participants were considered heavy drinkers if they consumed more than 30 g of alcohol per day^59^. We considered individuals to be engaging in a regular physical activity if they performed at least 20 min of high-intensity exercise three times per week or 30 min of moderate-intensity exercise five times per week. Income levels were separated into the bottom 20% and the rest.

Participants' baseline comorbidities were determined using their medical histories in conjunction with clinical and pharmacy codes of the ICD-10-CM. Comorbidities such as hypertension, diabetes, and dyslipidemia were adjusted according to the following criteria as described previously^58^. Hypertension was defined as getting antihypertensive drugs at least once a year under ICD-10-CM codes I10-I13, I15, or having a blood pressure of 140/90 mmHg or higher. Diabetes mellitus (DM) was defined as having a fasting glucose level of more than 126 mg/dL or getting a prescription for hypoglycemic drugs at least once a year under ICD-10-CM code E11-E14. Dyslipidemia was defined as having a total cholesterol level of 240 mg/dL or higher or as at least one claim per year for prescription of a lipid-lowering drug under ICD-10-CM code E78.

To define metabolic syndrome, we used the same criteria as described in a previous study^58^. Both modified criteria of the National Cholesterol Education Program Adult Treatment Panel III and modified waist circumference (WC) cutoff for Asians were used to define metabolic syndrome^60,61^. Individuals were considered to have metabolic syndrome if they possessed at least three of the following characteristics: (i) abdominal obesity, WC ≥ 90 cm for men or ≥ 85 cm for women; (ii) hypertriglyceridemia, serum triglycerides ≥ 1.70 mmol/L or treatment with lipid-lowering medication; (iii) low high-density lipoprotein (HDL), serum HDL-cholesterol < 1.04 mmol/L for men or < 1.30 mmol/L for women or treatment with lipid-lowering medication; (iv) hypertension, systolic blood pressure (BP) ≥ 130 mmHg, diastolic BP ≥ 85 mmHg, or treatment with antihypertensive medication; and (v) glucose intolerance, fasting plasma glucose ≥ 5.55 mmol/L or use of hypoglycemic agents.

### Definition of proteinuria

Proteinuria was determined using a urine dipstick test. With high sensitivity and specificity of more than 90%, the urine dipstick test can find proteinuria when the reference standard is ACR 300 mg/g.^62,63^ In the urine dipstick test, the degree of proteinuria was measured as negative, trace, 1 + , 2 + , 3 + , or 4 + according to proteinuria concentration-dependent color difference. The color of the dipstick, which ranged from negative to + 4, corresponded to the following urine protein concentrations: undetectable, 10 mg/dL, 30mg/dL, 100 mg/dL, 300mg/dL, and 1000 mg/dL, respectively.

The study population was classified into six groups according to the degree of proteinuria (negative, trace, 1 + , 2 + , 3 + , and 4 +). The eGFR was calculated using the following formula from the Modification of Diet in Renal Disease study: eGFR = 175 serum creatinine-1.154 age-0.203 0.70 (for women)^18,31^.

### Statistical analysis

For continuous variables, data are presented as mean and standard deviation. For categorical variables, data are presented as numbers and percentages. Baseline characteristics were compared according to levels of proteinuria using either analysis of variance (ANOVA) for continuous variables or chi-squared test for categorical variables. Baseline characteristics are primarily presented as mean ± standard deviation (SD). The incidence of OCNP was expressed as the incidence rate per 100,000 person-years by dividing the number of cases of OCNP by the total number of person-years. Using the Kaplan–Meier method and the log-rank test, the cumulative incidence of ONCP was compared by proteinuria level.

To examine the relationship between proteinuria and the risk of OCNP, we used the Cox proportional hazards model and reported results as HRs with 95% confidence intervals (CIs). To adjust for other potential confounding factors, we independently set up and compared four models. Model 1 was analyzed without adjustment. Model 2 was adjusted for gender and age. Model 3 was adjusted for gender, age, smoking status, alcohol consumption, regularity of physical activity, and income status. Model 4 was adjusted for gender, age, smoking status, alcohol consumption, regularity of physical activity, income status, metabolic syndrome, and eGFR. HR and CI were also determined to evaluate the risk of incidence of OCNP in relation to the level of proteinuria detected by a dipstick test. Statistical significance was considered if *p*-value was less than 0.05. All statistical analyses were conducted using SAS software version 9.3 (SAS Institute, Cary, NC, USA).

## Data Availability

The data used in the current study were obtained under a license from the National Health Insurance Sharing Service of Korea and are not publicly available. Data can be accessed by the corresponding author, Kyung-Ah Park, upon a reasonable request and with the permission of the National Health Insurance Sharing Service of Korea.
